# Re-inventing the future of the radiological research footprint in South Africa

**DOI:** 10.4102/sajr.v22i1.1665

**Published:** 2018-10-15

**Authors:** Maya Patel

**Affiliations:** 1 Radiological Society of South Africa, South Africa; 2 National Radiology Services Inc., Johannesburg, South Africa

The accidental discovery of the usefulness of radiation in medical diagnostics in 1895 has changed the way healthcare is delivered and is associated with tremendous growth in the field of radiology, mainly because of advances in research. Such advances have created a specialised and -sub-specialised discipline to such an extent that most clinicians rely on radiological imaging to confirm a diagnosis, monitor progress or predict an outcome for a patient. As a result, imaging is requested for almost every medical encounter.

Medical advances, both locally and internationally, have, however, far surpassed radiology, creating a new world, ranging from stem cell therapy to robotic and artificial intelligence, totally changing the landscape of practice. Analogously, our profession needs to foster and encourage further research if we wish to remain at the forefront of innovation. Cutting-edge radiology research should be driven and celebrated by everyone.

Admirably, the past two decades have witnessed a dramatic rise in radiology research in South Africa. This is attributed mostly to the requirement of a Master’s degree to fulfil university criteria for a postgraduate degree in radiology. Simultaneously, there has also been a fair amount of radiology PhD studies completed. This recent spark poses an interesting scenario where current research is highlighting the need for follow-up studies, protocols, policies and guidelines locally relevant to the South African context, but will it be enough to lead us into the rapidly changing future?

As in any developing country, the South African stage faces limitations. One of the major factors is the huge divide between public and private services, both in terms of human and physical resources. The reality is that the public sector environment is severely restricted in its ability to provide modern imaging modalities, the international forefront of radiological research; thus, specialised modalities in the form of Magnetic resonance imaging (MRI) and Positron emission tomography–computed tomography (PET/CT) are often only accessible in subspecialist- and specialist-level hospitals in South Africa. In addition, the large burden of disease increases the workload to such an extent that despite the disease profiles, offering a wealth of opportunities to conduct research, radiologists are often unable to dedicate the necessary time and energy required. Furthermore, staffing and expertise are limited, with skilled staff often seeking alternate opportunities in the more lucrative private environment. Consequently, the entire system presents an immense research challenge, ranging from infrastructural needs to availability of recorded data, loss to follow-up, lack of facilities, limited expertise and unavailability of several commercial medications and contrast agents. These local challenges are, however, not unique to radiologists and apply to all medical professions.

Within our small radiology community, the research quality is modest, focusing primarily on frequency statistics, retrospective data capture and auditing. Comparatively, we appear to be strides behind our international counterparts who are documenting novel concepts such as new 3D imaging virtual platforms and conducting prospective clinical research trials. Nevertheless, although basic, locally relevant research within our country’s limitations benefits both ourselves and other developing countries that may be facing similar challenges.

International guidelines are available, tested and established, but as mentioned, more and more studies are highlighting the need for locally relevant criteria and service delivery improvement. In this respect, the South African footprint is being embedded and for now it seems that we are making steady progress. Our research agenda should be driven in line with what is best for patient safety, efficacy in care and improved quality at a lower cost. This then incorporates the moral and ethical dimensions into research that is relevant to our context within South Africa and can inform improved care for our patient population, addressing the disease profiles that we face. At the same time, we need not to lose sight of the modern role of medical imaging, as seen internationally, and promote some attention on the evolution of innovation in technology around us, and specifically in radiology. In this way, we can create a profession that our patients expect and that the medical profession demands.

To achieve this, we need to lead research in conjunction with other medical teams. This agenda should become part of the strategy of the organisation for both the radiologists and other departments. Private and public sectors need to collaborate to diminish the divide and inequity, thereby strengthening the health service delivery as a whole. Such collaboration could also take place on the academic level, hence supporting research through offering assistance in terms of resources, infrastructure and expertise, while at the same time stimulating continuing professional development (CPD) for colleagues who are no longer exposed to the academic environment. Another initiative would be to review the process of undergraduate and postgraduate teaching by encouraging research at these levels and stimulating interest at an early stage.

Within academic publishing, a journal’s research footprint is used to track its scholarly and scientific impact, setting it against other journals and marking its position in the academic literature world. Although the impact of the journal is always in focus, the benefit that its research content offers will inevitably reflect a standard that can be shared and honoured. With the aid of research, we as radiologists can re-invent the future of radiology in our country and secure our place in the future of medicine. We certainly have the potential and the expertise to do so.

